# Problematic internet use and its associated factors among the students of higher secondary schools of Kathmandu Metropolitan City, Nepal

**DOI:** 10.1371/journal.pgph.0006511

**Published:** 2026-10-09

**Authors:** Manish Rajbanshi, Susmita Thapa, Richa Aryal, Jesika Joshi, Birendra Bishwokarma, Dinesh Raj Neupane, Sushan Man Shrestha, Durga Prasad Pahari

**Affiliations:** 1 Central Department of Public Health, Institute of Medicine, Tribhuvan University, Kathmandu, Nepal; 2 Department of Public Health and Community Medicine, Chitwan Medical College, Tribhuvan University, Chitwan, Nepal; 3 Department of Psychology, Padma Kanya Multiple Campus, Tribhuvan University, Kathmandu, Nepal; PLOS: Public Library of Science, UNITED STATES OF AMERICA

## Abstract

Problematic Internet Use (PIU) is a growing concern among adolescents in developing countries like Nepal due to increased access to electronic devices and the internet. Excessive use is associated with academic difficulties, inattention, and social isolation. Although it is an emerging concern, there is limited evidence on factors that contribute to PIU among students in Nepal. Thus, this study aimed to assess the prevalence of PIU and its associated factors among higher secondary school students of Kathmandu Metropolitan City (KMC). A cross-sectional study was conducted among 406 students in grades 11 and 12. Participants were selected using multistage random sampling. A self-administered questionnaire, the Internet Addiction Test (IAT), was used for data collection. Bivariate analysis was performed using the Chi-square test and bivariate binary logistic regression analysis. Multivariate logistic regression analysis was used to determine the factors associated with PIU. Variables with a p-value < 0.1 in bivariate analysis were included in the multivariate logistic regression model. The mean (±SD) age of the participants was 17.22 ± 1.03 years. This study found that 42.6% (95% CI: 37.0%-47.0%) of participants had PIU. Participants aged 19 years and above (AOR = 2.16, 95% CI:1.81-5.24), those from non-agricultural family income (AOR = 1.27, 95% CI: 1.07-2.25), and those who preferred YouTube (AOR = 1.70, 95% CI: 1.07-2.72), and Facebook (AOR = 1.65, 95% CI: 1.40-2.06) were at higher risk of PIU. Similarly, participants who used the internet in the evening (AOR = 2.01, 95% CI: 1.09-3.69), at night (AOR = 2.03, 95% CI: 1.21-3.38), and over 8 hours per day (AOR = 2.69, 95% CI: 1.48-4.91) had higher odds of PIU. This study revealed a high prevalence of PIU among adolescents. The study findings highlight the need for targeted interventions, including awareness programs, school-based preventive strategies and regulation of internet use time to promote healthy internet behavior among students.

## Introduction

Problematic Internet Use is an overwhelming and uncontrollable obsession with using computers and the internet, leading to significant problems in daily life, such as instability in mental and emotional state, impairment in occupational and social interactions [1]. It includes activities like gaming, watching videos, listening to music, online gambling, and social networking [2]. The common signs of PIU among students are obsession with the internet, irritability when offline, social isolation, poor school performance, sleep loss, and eye strain [3]. Access to the internet through mobile phones and tablets can lead to excessive use, impacting their personal, academic, and social lives [4]. Younger age groups (6–24 years) are especially vulnerable to PIU due to heavy social media use [3,5].

Globally, over 60% of people use social media, with an average daily usage of over two hours [6]. Adolescents spend more time on social media than other groups, with males more active than females [6]. Internet usage is highest in Eastern Asia (75%), followed by North America (71%), South America (68%), Southern Asia (32.9%), and West Africa (16%) [6]. The most used platforms worldwide are Facebook (3065 million), YouTube (2504 million), Instagram (2000 million), WhatsApp (2000 million), and TikTok (1582 million) [6]. Internet penetration is 86.6% in developed countries, compared to 47% in developing countries and 19.1% in least developed countries [3,6,7], rising from 17% in 2005 to 54% in 2019 in developed regions [6–9]. In Europe, the prevalence of PIU among adolescents ranges from 1.6% to 15%, while in Southeast Asia it shows a much wider range, from 1% to 47.4% [10,11].

In Nepal, over half the population uses the internet, 44.2% access it weekly, and about 70% of households in Kathmandu Valley have internet access [4]. In Nepal, particularly in urban areas like Kathmandu Metropolitan City (KMC), internet access among higher secondary school students has increased [4]. However, its use for academic purposes is not consistently structured or guided within school curricula, and internet use is often driven by individual preferences rather than formal educational integration [4,7]. As a result, students frequently rely on self-directed internet use, which may increase exposure to non-academic content [8]. In Nepal, 21.5% of adolescents show borderline and 13.3% show possible PIU [4]. Overall, around 35% of Nepalese adolescents are at risk of PIU [4,10,11].

Adolescents increasingly use the internet throughout the day and night for academic, social, entertainment, and communication purposes, indicating a global challenge to the health system of the countries [4,7,8]. This widespread and easy access to the internet in developing countries like Nepal increases the risk of PIU among adolescents [7–9]. Adolescents today spend over seven hours daily on screens, more than the time spent sleeping or attending school [12,13]. While internet use can support academic and vocational growth, excessive use is linked to poor nutritional, mental, and physical health, substance abuse, academic difficulties, social isolation, self-harm, suicidal tendencies, and low self-esteem [9,14–16]. Since, adolescents are more prone to internet addiction and school settings show the highest prevalence, urgent and targeted preventive strategies are essential, especially in urban areas like Kathmandu, where internet access is high [14–16].

It is important to investigate PIU among adolescents, particularly in low- and middle-income countries such as Nepal, where rapid expansion of smartphone ownership and internet access has substantially increased digital engagement [15]. Although PIU has been widely studied in high-income countries, the findings may not be directly applicable to Nepal due to differences in socio-cultural, educational, and technological contexts. Despite growing concerns about PIU, evidence from Nepal remains limited, particularly among higher secondary school students in urban settings such as KMC, where internet penetration is among the highest in the country. Existing Nepalese studies have primarily focused on undergraduate or college students and have largely examined the prevalence of PIU and its association with sleep quality, depression, anxiety, and selected socio-demographic characteristics [14–16]. However, relatively little attention has been given to internet-use behaviors, including preferred social media platforms, timing and duration of internet use, and contextual family characteristics such as the primary source of household income. Addressing these evidence gaps is essential for understanding the determinants of PIU among adolescents and for developing context-specific, school-based interventions and policies to promote healthy internet use in Nepal.

Higher secondary school students are at a crucial stage of personal and academic development, addressing PIU in this group can help improve their overall well-being and prospects. Kathmandu District provides a particularly relevant setting for investigating PIU because it is the most urbanized and digitally connected district in Nepal. Adolescents in Kathmandu have greater access to high-speed internet, smartphones, online gaming, and social media platforms than those in many other parts of the country. These factors may elevate the risk of developing problematic patterns of internet use.

Thus, this study aimed to assess the prevalence and factors associated with PIU among higher secondary school students of KMC. Understanding the magnitude and determinants of PIU among adolescents in Kathmandu is essential for developing evidence-based interventions tailored to the local context. The findings can inform policymakers, school administrators, parents, and public health professionals in designing school-based educational programs, strengthening digital literacy, promoting healthy internet use, and preventing internet-related behavioral problems. Furthermore, generating local evidence will contribute to the development of national strategies and policies addressing adolescent digital well-being in Nepal.

## Materials and methodology

### Study design and setting

This study followed a cross-sectional design and was conducted among higher secondary school students in KMC, Kathmandu district, Nepal. KMC is divided into 32 wards and has a literacy rate of 90.5% as per the 2021 Census [17]. A total of 470 higher secondary schools (HSS) are located within the city, comprising both government and private schools.

### Study population

The study population included students from grades 11 and 12 studying in government and private higher secondary schools within KMC. Students who had access to at least one internet-enabled device were eligible for this study. Students who were unable to provide written consent or schools with only grade 11 were excluded from the study.

### Sample size and sampling technique

The sample size was calculated using a Proportion Cochrane formula Z^2^pq/d^2^ (Cochrane et. al., 1977) [18]. The proportion of PIU among adolescents (P = 21.5%) was taken from a similar study conducted in Nepal by Karki K et al., 2020 [4].

Assuming, 95% Confidence Interval (CI), and 5% allowable error (d), and a 10% non-response rate, the sample size was determined as 271. After applying a design effect of 1.5, the minimum required sample size for this study was 406.

A multistage random sampling method was followed to select the participants. Altogether, there were 470 higher secondary schools in KMC. Private and government higher secondary schools (HSS) were considered as strata. Then, four government HSS and six private HSS were selected randomly using a lottery method. The objective of selecting four government and six private schools was to ensure representation of both school types rather than to achieve proportional allocation based on the total number of schools or students. Schools within each stratum were selected randomly using the lottery method

Again, grades 11 and 12 were considered as strata. In case of more than one section, one section from each of grades 11 and 12 was selected randomly. Initially, all students in each randomly selected section were invited to complete the questionnaire. A total of 496 questionnaires were distributed to all eligible students in the randomly selected sections of Grade 11 (10 sections) and Grade 12 (10 sections). As data collection was conducted in classroom settings during regular school hours, all 496 questionnaires were returned, yielding a 100% response rate.

Following data collection, questionnaires from each selected section were reviewed separately. Sixteen questionnaires were excluded because they were incomplete or did not meet the study eligibility criteria. The remaining valid questionnaires were assigned unique identification numbers to ensure accurate identification and prevent duplication. From each selected section, 21 valid questionnaires were then selected using a simple random sampling technique (lottery method). This procedure was applied consistently across all 20 selected sections, resulting in 42 participants from each school (21 from Grade 11 and 21 from Grade 12) and a final analytical sample of 406 participants. (S1 Fig)

### Data collection and techniques

Data were collected between October 6 and November 22, 2024. The Principal Investigator (PI) led the data collection process, which took place in a classroom environment. Before data collection, participants were informed about the study’s purpose and objectives. They also received an orientation and clear instructions on how to complete the questionnaire. The participants took approximately 10–15 minutes to complete the questionnaire. A structured, self-administered questionnaire available in both English and Nepali was used. Support letters were obtained from each participating school beforehand. The data collection was further supported by school teachers and members of the research team.

### Tools and measures

The Internet Addiction Test (IAT), developed and validated by Kimberly S. Young, was used to assess PIU among adolescents and young adults [19]. It is one of the most widely used instruments for measuring PIU worldwide [19]. The original IAT has demonstrated excellent internal consistency (Cronbach’s α = 0.90–0.93) and good test–retest reliability (r = 0.85). In the present study, the Nepali version of the IAT, translated and adapted by Gautam et al., was used to assess PIU among adolescents [19,20]. The instrument was translated into Nepali by bilingual experts and independently back-translated into English to ensure semantic equivalence with the original version [20]. It has been extensively applied in school-based populations, including higher secondary school students, to assess the extent of PIU. The IAT is a 20-item self-report questionnaire that measures characteristics and behaviors associated with compulsive internet use, including compulsivity, escapism, and dependency [19]. The study tool was pretested among 10% of the sample to assess internal consistency prior to data collection. The IAT demonstrated good reliability, with a Cronbach’s alpha of 0.86. Participants’ responses were measured on a 6-point Likert scale ranging from 0 to 5 (0 = does not apply, 1 = rarely, 2 = occasionally, 3 = frequently, 4 = often, and 5 = always) [19].

The study tool had three sections. The first section included information on the socio-demographic and household characteristics of the participants, such as age, sex, ethnicity, religion, academic class, educational faculty, marital status, family type, parental education, family monthly income, and main source of family income. “Main source of family income” refers to the primary source contributing to household earnings. “Agriculture” indicates income mainly from farming activities, while “non-agriculture” includes income from business, service, foreign employment, or other non-farming sources. The second section consisted of information related to the use of the internet, such as devices for internet use, the purpose of using the internet most of the time, the most used website, access to an internet connection (yes/no), duration of internet usage (in hours) and internet use time.

The third section consisted of information related to IAT such as time overuse, chore neglect, internet over friends, new online relations, complaints from others, academic impact, email before tasks, work productivity loss, online secrecy, escape through internet, online anticipation, fear without internet, annoyed when interrupted, sleep loss, offline preoccupation, few more minutes, failed reduction attempts, time hiding, prefer online over outing, and withdrawal symptoms [19].

To measure PIU, each item of IAT was rated on a 6-point Likert scale ranging from score 0 to 5, where ‘0’ indicated “not applicable,” ‘1’ “rarely,” ‘2’ “occasionally,” ‘3’ “frequently,” ‘4’ “often,” and ‘5’ “always” [19]. The total IAT score for each participant ranged from 0 to 100. Based on the standard interpretation of the IAT, scores below 40 indicate normal internet users, 40–69 indicate problematic internet users, and 70–100 indicate severely problematic internet users [19]. Participants with IAT scores ≥40 (problematic and severely problematic categories) were classified as having PIU [19,20].

### Data management and analysis

Collected data were cleaned, coded, filtered, and verified in EpiData software version 3.1 and exported to IBM Statistical Package for Social Science (SPSS) version 25. Individual characteristics were displayed using numbers, percentages, mean, and standard deviation. A Chi-square test and bivariate binary logistic regression were performed for bivariate analysis.

Multiple regression models were then constructed using different inclusion thresholds (variables with p-values < 0.1, 0.2, and 0.3) and compared with a full model based on theoretical relevance. Then, variables with p < 0.1 were included in the final multivariable model to adjust for potential confounding factors. Multi-collinearity was checked using the Variance Inflation Factor (VIF) among the variables before conducting multivariate regression analysis. Hosmer-Lemeshow Test, Nagelkerke R^2^ value and Omnibus test were considered for the goodness of the regression analysis model. All the tests were conducted at a 95% Confidence Interval (CI), and variables with a p-value <0.05 were considered statistically significant.

### Ethical consideration

This study was reviewed and approved by the Institutional Review Committee (IRC) of the Institute of Medicine, Tribhuvan University, Nepal [Reference No: 220 (6-11) E2081/82]. A letter of support was obtained from the Kathmandu Metropolitan City Office [Reference No: 339] for conducting the study. Written permission was obtained from each selected school prior to data collection. Written informed consent was obtained from each participant, and for those under 18 years of age, written consent was obtained from their parents or caretakers one day before data collection. The objectives and purpose of the study were clearly explained to the participants before data collection. The anonymity and confidentiality of participants were strictly maintained throughout the study. Participants were also informed of their right to withdraw from the study at any stage without any negative consequences.

## Results

### Socio-economic and demographical characteristics of the participants

The mean (±SD) age of the participants was 17.22 ± 1.03 years. Approximately, half of the participants were male (50.5%). Most participants belonged to the Janajati (45.8%) ethnicity and lived in nuclear families (52.7%). Three-fourths of them were enrolled in private schools (74.9%). The majority of participants reported having good relationships with their parents (94.1%) and friends (89.2%) (Table 1).

**Table 1:** Socio-economic and demographic characteristics of the participants.

Characteristics	Number (n)	Percentage (%)
**Age (in years)**		
**Mean ± SD**	17.22 ± 1.03	
≤ 16	104	25.6
17	134	33.0
18	140	34.5
≥ 19	28	6.9
**Sex**		
Female	201	49.5
Male	205	50.5
**Ethnicity**		
Brahmin	84	20.8
Chhetri	95	23.5
Janajati	186	45.9
Madhesi	11	2.8
Muslim	6	1.5
Dalit	22	5.5
**Religion**		
Hindu	314	77.4
Buddhist	64	15.8
Christian	16	3.9
Islam	12	2.9
**School type**		
Private	304	74.9
Government	102	25.1
**Educational background**		
Administration/management	150	36.9
Science	127	31.3
Hotel management	96	23.6
Humanities	33	8.2
**Marital status**		
Married	6	1.5
Unmarried	400	98.5
**Family type**		
Nuclear	214	52.7
Joint	176	43.4
Extended	16	3.9
**Mothers’ education**		
Cannot read or write	92	22.7
Can read and write	157	38.7
Primary level	81	20.0
Secondary level	57	14.0
Bachelor	16	3.9
Master level and above	3	0.7
**Fathers’ education**		
Cannot read and write	22	5.4
Can read and write	142	35.0
Primary level	65	16.0
Secondary level	117	28.9
Bachelor	44	10.8
Master level and above	16	3.9
**The main source of income in a family**		
Agriculture	120	29.7
Business	117	29.0
Foreign employment	82	20.3
Service	50	12.4
Labour/wages	26	6.4
Others (INGOs, NGOs)	11	2.2
**The monthly income of a family (in USD)**		
≤ NRs 25000 (≤USD 182.5)	42	10.3
NRs 26000–50000 (USD 189.8-365)	217	53.4
NRs 50000 and above (≥ USD 365)	147	36.3
**Relationship status with parents (self-reported)**		
Good	382	94.1
Not good	24	5.9
**Relationship status with friends (self-reported)**		
Good	362	89.2
Not good	44	10.8

### Internet-related characteristics of the participants

Most participants had access to mobile phones (88.2%), and laptops (38.4%) for internet use. Internet usage was primarily for entertainment (53.7%) and acquiring knowledge (39.2%). Social media such as YouTube (49.5%) and Instagram (39.2%) were the most used. Only a small proportion of participants (17.5%) reported using the internet for over 8 hours per day. Evening (33.0%) and night (42.9%) were the preferred times for internet use (Table 2).

**Table 2:** Internet-related characteristics of the participants.

Characteristics	Number (n)	Percentage (%)
Access to a device for Internet use*		
Mobile	358	88.2
Laptop	156	38.4
Smartphone	141	34.7
Desktop/computer	48	11.8
Tablet	35	8.6
Purpose of Internet use*		
Entertainment	218	53.7
Acquiring knowledge	159	39.2
Social networking/communication	149	36.7
Website most used*		
YouTube	201	49.5
Instagram	159	39.2
Facebook	120	29.6
Google	68	16.7
Tiktok	61	15.0
**Availability of Wi-Fi at home**		
Yes	354	87.2
No	52	12.8
**Duration of internet use daily (in hours)**		
**Mean ± SD**	**5.0 ± 3.2**	
Below 8	335	82.5
8 and above	71	17.5
**The most preferred time for Internet use**		
Night	174	42.9
Evening	134	33.0
Afternoon	90	22.1
Morning	8	2.0

*multiple-response questions.

### Prevalence of PIU among the participants

This study found that 42.6% of participants were classified as having PIU, while 57.4% were normal internet user (Table 3).

**Table 3:** Prevalence of PIU among the participants.

Internet Use Category	Number (n)	Percentage (%)	P*, 95% CI
Normal Internet user	233	57.4	0.57 (0.52-0.62)
Problematic Internet use	173	42.6	0.42 (0.37-0.47)

P* Proportion.

### Association between individual characteristics and PIU

Students aged 19 years or older had 2.16 times higher odds of being problematic internet users compared with those aged less than 19 years (AOR = 2.16, 95% CI: 1.81–5.24, *p* = 0.037). Similarly, students from families whose main source of income was non-agriculture had 1.27 times higher odds of problematic internet user than those from families primarily dependent on agriculture (AOR = 1.27, 95% CI: 1.07–2.25, *p* = 0.031) (Table 4).

**Table 4:** Association between individual characteristics and PIU.

Characteristics	Problematic Internet User		p-value	Crude Odds Ratio (COR) 95% CI	p-value	Adjusted Odds Ratio (AOR)
	Yes	No				
	n (%)	n (%)				
**Age**						
< 19	157 (41.5)	221 (58.5)	0.043	Ref	0.037	Ref
≥ 19	16 (57.1)	12 (42.9)		1.87 (1.12–2.58)		2.16 (1.81–5.24)
**Sex**						
Male	91 (44.4)	114 (55.6)		1.15 (0.78–1.71)		
Female	82 (40.8)	119 (59.2)	0.483	Ref		
**Ethnicity**						
Brahmin	30 (35.7)	54 (64.3)		1.43 (0.35–5.83)		
Chhetri	42 (44.2)	53 (55.8)		1.50 (0.42–5.33)		
Janajati	85 (45.7)	101(54.3)		1.05 (0.30–3.68)		
Madhesi and Muslim	11 (36.7)	19 (63.3)		0.99 (0.29–3.35)		
Dalit	5 (45.5)	6 (54.5)	0.573	Ref		
**School type**						
Government	37 (36.3)	65 (63.7)		0.70 (0.24–0.81)	0.880	0.94 (0.43–2.03)
Private	136 (44.7)	168 (55.3)	0.033	Ref		Ref
**Educational background**						
Science	60 (47.2)	67 (52.8)		0.82 (0.38–1.78)		0.94 (0.50–1.76)
Administration/management	52 (34.7)	98 (65.3)		1.38 (0.64–2.99)		1.23 (0.39–3.89)
Hotel management	47 (49.0)	49 (51.0)		0.76 (0.34–1.70)		1.79 (0.90–3.53)
Humanities	14 (42.4)	19 (57.6)	0.089	Ref	0.192	Ref
**Family type**						
Nuclear	95 (44.4)	119 (55.6)		1.16 (0.78–1.73)		
Joint/extended	78 (40.6)	114 (59.4)	0.482	Ref		
**Mother education**						
Can read and write	66 (42.0)	91 (58.0)		1.06 (0.63–1.78)		
Primary level and above	67 (42.7)	90 (57.3)		1.30 (0.71–2.40)		
Cannot read and write	40 (43.5)	52 (56.5)	0.529	Ref		
**Father education**						
Can read and write	58 (40.8)	84 (59.2)		1.07 (0.40–2.49)		
Primary level and above	106 (43.8)	136 (56.2)		0.88 (0.36–2.15)		
Cannot read and write	9 (40.9)	13 (59.1)	0.841	Ref		
**The main source of income in a family**						
Non-agriculture	132 (46.2)	154 (53.8)		1.65 (1.23–2.44)		1.27 (1.07–2.25)
Agriculture	41 (34.2)	79 (65.8)	0.026	Ref	0.031	Ref
**The monthly income of the family**						
Below 25000	19 (45.2)	23 (54.8)		1.21 (0.62–2.35)		
26000-50000	88 (40.8)	129 (59.4)		1.01 (0.50–2.01)		
50000 and above	66 (44.9)	81 (55.1)	0.688	Ref		
Median (IQR)	50000 (65000–300000)					
**Relationship status with parents**						
Good	160 (41.9)	222(58.1)		0.61 (0.26–1.39)		0.75 (0.24–1.65)
Not good	13 (54.2)	11 (45.8)	0.028	Ref	0.347	Ref
**Relationship status with family**						
Good	151 (41.7)	211(58.3)		0.71 (0.38–1.33)		
Not good	22 (50.0)	22 (50.0)	0.334	Ref		

Hosmer-Lemeshow model of fitness =0.692, Nagelkerke R^2^=0.265.

Omnibus test = p < 0.001.

### Association between Internet-related characteristics and PIU

Students who used the internet for 8 hours or more per day had significantly higher odds of PIU compared with those using it for less than 8 hours per day (AOR = 2.69, 95% CI: 1.48–4.91; *p* = 0.001). Compared with students who primarily used the internet in the morning, those who most frequently used the internet at night had approximately two times higher odds of PIU (AOR = 2.03, 95% CI: 1.21–3.38), while those who primarily used it in the evening also had about twice the odds (AOR = 2.01, 95% CI: 1.09–3.69). Students who reported YouTube as their most frequently used website had significantly higher odds of PIU than those who primarily used TikTok (AOR = 1.70, 95% CI: 1.07–2.72) (Table 5).

**Table 5:** Association between Internet-related characteristics and PIU.

Characteristics	Problematic Internet User		p-value	Crude Odds Ratio (COR) 95% CI	p- value	Adjusted Odds Ratio (AOR) 95% CI
	Yes	No				
	n (%)	n (%)				
Access to a device for Internet use*						
Mobile	155 (43.3)	203 (56.7)		1.27 (0.68–2.36)		0.72 (0.92–2.60)
Laptop	71 (45.5)	85 (54.5)		1.21 (0.80–1.81)		0.92 (0.42–2.36)
Tablet	21 (60.0)	14 (40.0)		2.16 (1.06–4.38)		0.84 (0.33–2.15)
Desktop/computer	26 (54.2)	22 (45.8)		1.69 (1.22–3.10)		0.75 (0.33–1.72)
Smartphone	67 (47.5)	74 (53.5)	0.051	Ref	0.096	Ref
Purpose of Internet Use*						
Entertainment	96 (44.0)	122 (56.0)		1.13 (0.76–1.68)		1.19 (0.94–2.47)
Social Networking	66 (45.2)	80 (54.8)		1.18 (0.78–1.77)		1.52 (0.94–2.47)
Acquiring Knowledge	54 (34.0)	105 (66.0)	0.005	Ref	0.084	Ref
Website most used*						
YouTube	71 (35.3)	130 (64.7)		0.55 (0.37–0.82)		1.70 (1.07–2.72)
Facebook	61 (50.8)	59 (49.2)		1.16 (1.04–2.56)		1.65 (1.40–2.06)
Google	35 (51.5)	33 (48.5)		1.54 (0.91–2.61)		1.21 (0.85–3.16)
Instagram	78 (49.1)	81 (50.9)		1.54 (1.03–2.30)		0.93 (0.57–1.52)
Tiktok	27 (44.3)	34 (55.7)	0.037	Ref	0.058	Ref
**Availability of Wi-Fi at home**						
No	23 (44.2)	29 (55.8)		0.92 (0.51–1.6)		
Yes	150 (42.4)	204 (57.6)	0.881	Ref		
**Duration of Internet use daily (in hours)**						
≥ 8	47 (66.2)	24 (33.8)		Ref		Ref
< 8	126 (37.6)	209 (62.4)	0.000	3.24 (1.89–5.56)	0.001	2.69 (1.48–4.91)
**The most preferred time for Internet use**						
Night	92 (52.9)	82 (47.1)		1.53 (1.12–2.30)		2.03 (1.21–3.38)
Evening	46 (34.3)	88 (65.7)		1.14 (1.26–5.01)		2.01 (1.09–3.69)
Afternoon	32 (35.6)	58 (64.4)		1.08 (1.24–4.85)		1.97 (0.42–9.20)
Morning	3 (37.5)	5 (62.5)	0.005	Ref	0.007	Ref

*Multiple response questions.

Hosmer-Lemeshow model of fitness =0.692, Nagelkerke R^2^=0.265.

Omnibus test = p < 0.001.

## Discussion

This study showed that a notable proportion of the students had PIU (42.6%) which is similar to the studies conducted in Nepal by Karki et. al (35%) [4], Acharya et. al (30%) [21], and Bhandari P.M et. al (36%) [22], Jordan (40%) [23] and Iran (39.6%) [24]. The higher proportion of PIU is due to increased access and availability of internet devices and higher internet in Nepal, especially in the Kathmandu valley [2,6,7]. Another reason could be that adolescents and young adults are at a critical developmental stage, characterized by risk-taking behavior and susceptibility to peer influence, increasing vulnerability to PIU, sleep disorders, and gaming disorder [25]. These findings are also supported by the Meta-analysis conducted on Internet Gaming Disorder among students [10], a study conducted in Iran (41.3%) [24] and Israel (30.2%) [26]. In contrast, this study’s finding is lower than that reported in Ethiopia (80%) among undergraduate students [27].

This study demonstrated that students aged 19 years and above were at a greater risk of PIU, and this is supported by the similar study conducted in China [28]. This may be because students use the internet to cope with sleep disorders and to establish social relationships [29]. Additionally, their limited awareness of the consequences of PIU makes them less likely to set healthy boundaries [10,30,31]. Meanwhile, younger adolescents may struggle with self-regulation, making them more susceptible to addictive behaviors [1,30].

In bivariate analysis, students who belonged to private schools had higher odds of PIU than government school students. This finding is consistent with a similar study conducted in Nepal [22]. It might be due to students in private schools often belong to families with higher socio-economic status, giving them easier access to smartphones, laptops, and high-speed internet. Thus, all these factors contribute to excessive internet use among students.

This study revealed that students from families with non-agricultural sources of income had higher odds of PIU. It may be due to most of the work, such as business and service, needing excessive time due to workload, leaving parents with little time to monitor or engage with their children. This lack of supervision may lead to excessive internet use. However, this result is not coherent with the study conducted in Nepal, showing no association between the income source of the family and PIU [4,21].

In bivariate analysis, this study demonstrated lower odds of PIU among those who reported having a good relationship with their parents and friends than those who did not. This result is consistent with the study conducted in Nepal and China [21,28]. A good relationship promotes social interaction and bonding, preventing excessive internet use [22,25,32]. Another reason is that good interpersonal communication could be the source of information sharing and dissemination that regulates the appropriate internet use and gaming disorder among individuals.

In bivariate analysis, those students who had access to the internet using devices such as laptops/computers, mobile phones, and smartphones were at a greater risk of PIU. This is similar to the studies conducted in India [8], Ethiopia [27], and Bangladesh [33]. Laptops and mobile phones provide convenient access to the internet, enabling adolescents to stay online, blogging, gaming online, and social interactions [1,27]. Furthermore, adolescents may use the internet to escape from stress, boredom, or real-life challenges. Similar to the other studies conducted in Nepal [5,21], India [8], and Ethiopia [27].

In bivariate analysis, this study reported a higher odds of PIU among students who used the internet for social networking, online communication, gaming, searching for information, and entertainment purposes. Although a considerable proportion of students reported using the internet for acquiring knowledge (39.2%), the higher use for entertainment (53.7%) suggests that academic use of the internet may not be strongly guided or emphasized within schools. This indicates that students may rely more on self-directed use rather than structured academic engagement, which could contribute to problematic usage patterns [2,6,7].

The students who used social media such as YouTube and Facebook were at a high risk of PIU. In this study, most of the students used social media such as YouTube (49.5%), Instagram (39.2%), and Facebook (29.6%), which is higher than the global pooled prevalence of social media addiction (18.4%) among students. These findings highlight the need for target-based intervention to be carried out in school settings to prevent social media and PIU, especially among developing countries like Nepal.

Students who used the internet for 8 hours or more per day had significantly higher odds of PIU than those who used it for less than 8 hours per day. This finding is consistent with a similar study conducted in Nepal [21], Ethiopia [27], India [8], and Bangladesh [33]. Although prolonged internet use may increase exposure to online activities associated with PIU and reduce engagement in offline activities, the cross-sectional design of this study does not permit conclusions about the direction of this relationship [4,7]. It is also possible that students with PIU naturally spend more time online. Longitudinal studies are needed to clarify the temporal relationship between daily internet use and PIU.

This study found that students who primarily used the internet during the evening and at night had higher odds of PIU than those who mainly used it in the morning. Similar patterns have been reported in previous studies [34]. One possible explanation is that evening and night time internet use may coincide with greater privacy and reduced parental supervision, which could facilitate longer or less regulated online activities. However, because this study employed a cross-sectional design, the temporal direction of this association cannot be determined. It is equally plausible that adolescents with PIU are more likely to remain online during the evening and nighttime. Therefore, these findings should be interpreted as associations rather than evidence of a causal relationship.

## Conclusion

This study found a high prevalence (42.6%) of problematic internet use among higher secondary school students in Kathmandu Metropolitan City, indicating that problematic internet use is an important public health concern in this population. Older age (≥19 years), non-agricultural family income source, using YouTube as the most frequently accessed website, spending 8 hours or more per day on the internet, and preferring to use the internet in the evening and at night were associated with problematic internet use.

These findings highlight the need for school-based interventions that promote healthy digital behaviors, increase awareness of responsible internet use, encourage balanced online and offline activities, and strengthen parental and school support for adolescents.

### Strength and limitation

The study used a validated and widely accepted tool to assess PIU. Being conducted in Kathmandu Metropolitan City, the capital and an urban center of Nepal with a large and diverse student population, the study reflects a setting with varied socio-cultural backgrounds and high internet accessibility. The use of multistage random sampling among higher secondary school students in Kathmandu Metropolitan City improves the representativeness of the study population within the selected setting. Both English and Nepali versions of the questionnaire were used for data collection.

However, several limitations should be considered when interpreting the findings. First, because the study was conducted among higher secondary school students in an urban setting and included only students with access to at least one internet-enabled device, the findings may not be generalizable to adolescents in rural areas or to those without internet access. Second, although multistage random sampling was employed, selecting an equal number of valid questionnaires from each selected section may have introduced some sampling bias by not reflecting the natural variation in section size. Third, the cross-sectional design precludes establishing temporal or causal relationships between internet-related characteristics and problematic internet use, and reverse causation cannot be ruled out. Fourth, the use of a self-administered questionnaire may have introduced recall bias, information bias, and social desirability bias, potentially leading to underreporting of internet-use behaviors. Finally, problematic and severe problematic internet use were combined into a single outcome category because the number of participants with severe problematic internet use was too small for reliable subgroup analysis. Consequently, differences across severity levels of problematic internet use could not be examined.

## Supporting information

S1 Dataset Data for analysis. (XLSX)

S1 Fig Sampling framework. (TIF)
